# Dental Education Again

**Published:** 1884-04

**Authors:** 


### Editorial.

### DENTAL EDUCATION AGAIN.

This cannot be called a hackneyed subject, notwithstanding so much has been said and written upon it, for its importance warrants almost any amount of intelligent discussion. Dentistry is yet in a comparatively formative state, and it behooves all who love their profession to thoughtfully consider what its best interests demand. In England professional education has not even yet been reduced to a system, and the tendency there seems toward a divergence from that in existence here. America took the lead in the establishment of dental schools, but their character was largely determined by the then existing circumstances. When Harris proposed to establish chairs of dental professorship in medical colleges, he was met with a cold rebuff, and this was the reason for the organization of distinct dental colleges. The far-seeing men of that day recognized the possibilities that lay in the future of dentistry. A professional education was an imperative necessity, and if it could not be obtained in existing colleges, there was no way but to establish new ones. The rapid strides that dentistry was making, and the high scientific and professional ground that its best exemplars took, at last opened the eyes of medical men, and of late years the best of the medical schools have thrown wide their doors, and afforded facilities for the proper study of dental science. These have now so multiplied that, with the segregated dental schools, there has arisen a sharp competition for students, which instead of raising has lowered the educational standard. The whole matter rests upon a purely business foundation, and is quite beyond the control of professional ethics. Dental college authorities are responsible to no one, and practically graduate whom they please. When this power rests in the hands of high-minded men, those who have some regard for the ethics of professional life, it can work no harm. But with the multiplication of schools, the ambition of unqualified or unprincipled men to obtain a position, either for the supposed honor that it may bring them or the money which they hope to obtain from it, our schools are in danger of becoming mere diploma mills. Indeed, some of them are little more than that now. It is time that the profession left off the bickerings over mere matters of detail, such as have usually been discussed in our societies under the head of "Dental Education," and begin seriously to consider the general subject of an educational *system.*

In an editorial article in the number of the INDEPENDENT PRACTITIONER for December, 1883, we detailed some of the abuses of the present lack of system, and the editor of *The Southern Dental Journal* takes us to task for not openly naming the guilty college. The time is not yet ripe for individual attacks. Sufficient testimony has not yet been collected, nor is the profession yet fully awake to the evils that will surely flow from some of the practices alluded to. The naming of one college that was graduating unworthy or unqualified students would only arouse personal feeling, and bring the accusation of a desire to compass unworthy and ulterior objects. Besides, as has been done before, it would be asserted that the accused college was only doing that of which other institutions were guilty. But the time will come when, with abundant proof to substantiate them, these charges can be brought home with a force that cannot be resisted or evaded.

It is not long since a regularly chartered college in Wisconsin publicly offered its diplomas for sale to any one who chose to invest an insignificant sum. How was this announcement met by the profession? Surely, one would imagine that such a storm of indignation would be raised among those who had a particle of professional pride, that all dentistry would be in a tumultuous uproar. It scarcely made a ripple upon the surface. A few weak protests were made, seemingly for form's sake, but the disgraceful business was allowed to flourish, practically unmolested. The men engaged in the dirty traffic were simply doing openly what some other schools had done under cover of a form. They granted diplomas without an examination. Others had done the same thing after an examination that was but a burlesque.

Fraudulent colleges can no longer do any special harm in the State of New York, as in some other States, because the law gives to the State Society the power to stamp them as unworthy by refusing to extend recognition to them, in which case their diplomas are not only worthless, but are testimony against the practitioner who uses them. But what shall be done to the recognized colleges that graduate unqualified students? They are too well skilled in artifice to be readily caught. Ingenious methods have been devised whereby the ethical law may be kept in the letter, while violated in spirit. Within the past month a "student" made us a visit, and unwittingly revealed one of these tricks. He is matriculated at a dental college, but has never yet visited it. Constructively he is now supposed to be attending lectures, while he has never even been in the city in which the college is located. Next winter he expects to attend the last half of the term *as a second-course student*, and in the spring of '85 to graduate with all the honors attendant upon an extended term of study. Where is this thing to end if the competition among the colleges continues? What honor will there be in the possession of the D. D. S. if this prostitution of college authority be not checked? Already England looks with so much of suspicion upon our distinctive degree that the diplomas of but two of our universities are acknowledged there, and the special degree of Doctor of Dental Surgery is scarce a mark of distinction. It is idle to talk of obtaining the forfeiture of charters by legal process. Shrewd men will still keep on the windy side of the law, and the abrogation of a charter is more easily talked of than accomplished, as the medical profession learned in fighting the Buchanan schools.

The evils, present and prospective, of our want of educational system arise, to our apprehension, from the lack of responsibility on the part of the colleges. In the State of New York the literary schools and colleges are amenable to a Board of Regents, and this secures some kind of uniformity and regularity. The medical schools are practically unhampered, and the profession is becoming aroused to the necessity of placing some kind of check upon the wholesale graduation of uneducated boys. A bill has been offered in the legislature, providing for a State Board of Examiners, who alone shall have authority to grant diplomas in medicine. Of course it is opposed by many of the colleges, and another emasculated bill has been offered, presumably in their interests. But it is confidently expected that the Erie County Bill, as the original is termed, because of its having its origin in Buffalo, Erie County, will in the near future become a law. Other States have already secured such an one. In Illinois, Missouri, and other States, such a law seems to be working good to medicine.

But that profession labors under embarrassments that would be unknown to dentistry, because of the different and warring schools into which medicine is divided. Allopath, Homeopath and Eclectic could scarcely be expected to agree in anything, yet it is not claimed in the States already in possession of a State Board for the licensing of physicians that injustice is done to either.

Why should not dental colleges be made amenable to such a law? There is something monstrous in the idea of any man or set of men being allowed to license their own pupils. They cannot, from the very nature of things, be dispassionate and unprejudiced judges of the qualifications of those whom they themselves profess to have fitted for their vocation. The power should rest somewhere else than in the hands of the professors of the schools. Competent men should be appointed by State authority to examine candidates for graduation, and to establish a uniform system ; to set up an unvarying standard of qualification to which every student must attain before he can become possessed of a diploma, or a license to practice. When this is done there will be a stimulus to the acquisition of knowledge, and the pupil who simply goes to college to put in the requisite time may be distinguished from him who makes the most of the advantages which a college course presents. With such a Board, the time passed in college will be a matter of secondary consideration, and diplomas may be consistently granted to the possessor of the necessary knowledge, no matter where he may have obtained it. Such a Board will have no pecuniary interest in the graduation of students, and hence there will be no temptation to launch upon a suffering community a horde of unqualified pretenders to professional knowledge.

At present, the time standard is the only one the profession can depend upon. We can insist that no pupil shall be graduated who has not passed through a prescribed curriculum, and this, if it be not evaded, secures a certain amount of opportunity for study, although it is a very uncertain criterion by which to judge of the actual attainments of a student. Certain colleges have clamored for the destruction of even this feeble barrier to the indiscriminate granting of diplomas, but any thinking man will readily appreciate the abuses that would inevitably creep in, were every honest or dishonest professor in a dental college free to graduate whom he would, himself the only judge of the qualifications necessary. Too often, it might be, the standard erected would be the ability to pay the liberal fee exacted. We commend this subject to the consideration of thoughtful men.

